# A Comprehensive Review on Long vs. Short Regimens in Multidrug-Resistant Tuberculosis (MDR-TB) Under Programmatic Management of Drug-Resistant Tuberculosis (PMDT)

**DOI:** 10.7759/cureus.52706

**Published:** 2024-01-22

**Authors:** Ashwin Karnan, Ulhas Jadhav, Babaji Ghewade, Anjana Ledwani, Poorna Shivashankar

**Affiliations:** 1 Respiratory Medicine, Jawaharlal Nehru Medical College, Wardha, IND

**Keywords:** healthcare program optimization, treatment efficacy, short regimen, long regimen, programmatic management of drug-resistant tuberculosis (pmdt), multidrug-resistant tuberculosis (mdr-tb)

## Abstract

This comprehensive review delves into the intricate landscape of multidrug-resistant tuberculosis (MDR-TB) treatment within the programmatic management of drug-resistant tuberculosis (PMDT) framework. MDR-TB poses a substantial global health threat, necessitating targeted approaches for effective management. The analysis explores the historical evolution, efficacy, safety profiles, and implementation challenges associated with long and short regimens. The findings underscore the importance of individualized clinical practices, considering patient-specific factors, and the need for ongoing monitoring within PMDT programs. Recommendations advocate for integrating advanced diagnostics, continuous surveillance, and training for healthcare professionals. The review concludes with a nuanced outlook on long versus short regimens, emphasizing a balanced approach and the imperative role of collaborative efforts in shaping the future of MDR-TB treatment. This synthesis contributes to the ongoing discourse, providing valuable insights for healthcare practitioners, policymakers, and researchers working toward optimizing outcomes for individuals afflicted with MDR-TB.

## Introduction and background

Multidrug-resistant tuberculosis (MDR-TB) remains a significant global health challenge, posing a formidable threat to tuberculosis (TB) control efforts. As a form of TB caused by strains of Mycobacterium tuberculosis resistant to both isoniazid and rifampicin, MDR-TB necessitates specialized and targeted treatment approaches. The complexity of MDR-TB management has led to the establishment of programmatic management of drug-resistant tuberculosis (PMDT) strategies to enhance the effectiveness of treatment programs [1]. MDR-TB arises due to the misuse or inadequate administration of anti-TB medications, leading to the development of resistance to the two most potent first-line drugs: isoniazid and rifampicin. This resistance significantly complicates the treatment landscape, requiring alternative and often more prolonged regimens. The World Health Organization (WHO) estimates that nearly half a million new cases of MDR-TB emerge each year, underscoring the urgency and gravity of this public health issue [2].

PMDT represents a structured and comprehensive approach to address the challenges posed by MDR-TB. PMDT involves the integration of diagnostic, treatment, and support services within existing national TB control programs. This approach aims to improve the quality and accessibility of care for individuals with drug-resistant TB, ensuring standardized and effective management [3]. PMDT encompasses diagnostic tools, treatment protocols, and supportive measures tailored to the complexities of MDR-TB. It acknowledges the importance of a systematic and programmatic approach to tackle the intricacies of drug resistance, emphasizing the need for collaboration between healthcare providers, laboratories, and community-based organizations [3].

The purpose of this review is to critically examine the existing knowledge surrounding the treatment of MDR-TB, with a particular focus on comparing long and short regimens under the umbrella of PMDT. By delving into the historical evolution, efficacy, safety profiles, and implementation challenges associated with both long and short regimens, this review aims to provide a comprehensive understanding of the strengths and limitations of each approach. As MDR-TB continues to be a significant global health concern, the insights gained from this review may inform healthcare practitioners, policymakers, and researchers in optimizing treatment strategies and refining PMDT programs. By synthesizing current evidence and identifying research gaps, this review contributes to the ongoing discourse on improving outcomes for individuals afflicted with MDR-TB.

## Review

Long regimens in MDR-TB treatment

Definition and Components of Long Regimens

Extended treatment regimens for MDR-TB refer to those employed in the management of multidrug-resistant and rifampicin-resistant tuberculosis (MDR/RR-TB), typically spanning 18 months or more. These regimens can either adhere to standardized protocols or be individualized, with the selection of medications following a prioritized order as per revised guidelines [4,5]. These extended MDR-TB regimens should ideally range from 18 to 20 months, with modifications contingent upon patient response [6]. Core components of longer regimens usually involve the incorporation of a minimum of four effective drugs during the initial six months, followed by three drugs thereafter, and, under specific circumstances, the potential initiation of five drugs initially. Injectable aminoglycosides, later-generation fluoroquinolones, and other second-line drugs may be integral components of these regimens, with the intensive phase lasting six to seven months when an injectable agent is included [6,7]. Noteworthy within longer MDR-TB regimens is the BPaL regimen, a groundbreaking treatment lasting six to nine months, comprising bedaquiline, pretomanid, and linezolid [7].

Historical Perspective and Evolution of Long Regimens

The evolution of longer regimens for treating MDR-TB has been marked by significant changes. The conventional longer treatment regimen (LTR) for MDR-TB, as endorsed by the World Health Organization (WHO) in 2011, featured an intensive phase lasting eight months, with a total treatment duration of 20 months [4,8]. WHO recommendations for longer regimens emphasize the option for standardization or individualization, emphasizing the need for monitoring through cultures rather than relying solely on clinical parameters [4]. The key components of longer regimens typically involve the inclusion of a minimum of four effective drugs during the initial six months, followed by three drugs thereafter, with the consideration of initiating five drugs initially under specific circumstances [6].

While the availability of new and repurposed medications has facilitated the adoption of shorter, all-oral MDR-TB treatments, challenges persist in procurement, availability, and monitoring of clinical outcomes during the implementation of these regimens [7]. Integral to longer MDR-TB regimens is the BPaL regimen, a novel treatment course spanning six to nine months, comprising bedaquiline, pretomanid, and linezolid [8]. As the landscape of MDR-TB treatment continues to evolve, the balance between conventional longer regimens and innovative approaches like BPaL remains a critical consideration in addressing the challenges associated with MDR-TB.

Efficacy of Long Regimens in Clinical Trials

The longstanding paradigm of employing longer regimens for the treatment of MDR-TB has been firmly established as the standard of care over the years. The efficacy of these regimens has been substantiated through various clinical trials, prompting the World Health Organization (WHO) to endorse individualized longer regimens for MDR-TB treatment [8]. Emphasizing the need for flexibility, the recommended total duration of these regimens spans from 18 to 20 months, adaptable based on patient response [7].

A comprehensive case-control study revealed that the total treatment duration significantly influences relapse rates, indicating that more extensive disease necessitates prolonged treatment, and fewer total doses heighten the risk of inadequate treatment [9]. While the DOTS guidelines prescribe a short course regimen for newly diagnosed patients, characterized by an initial intensive phase of four drugs for two months followed by a continuation phase of rifampicin and isoniazid for four months, an intriguing finding from the study suggests that the extension of treatment is protective against relapse [9].

Contrastingly, the groundbreaking STREAM trial demonstrated that a nine to 11-month “short” regimen exhibited non-inferior efficacy and comparable safety to a 20+ month “long” regimen [10]. However, analysis at week 132 revealed that 84.2% and 83.1% of patients in the short and long regimens achieved cure and completed treatment, even if they had experienced treatment failure or relapse, necessitating modifications or restart of treatment [10]. This raises the possibility that longer regimens may yield superior treatment completion and relapse prevention outcomes.

In a phase III randomized controlled trial, three new drug regimens demonstrated efficacy and safety comparable to conventional treatments while reducing treatment duration [11]. These regimens hold promise as alternative options for patients unable to receive other recommended treatments. However, the trial's results underscore the need to eliminate barriers hindering timely and high-quality care for MDR-TB treatment [11]. In summary, the efficacy of longer regimens for MDR-TB treatment is well-established. While shorter treatment regimens (STR) show non-inferiority, longer regimens may offer advantages regarding treatment completion and relapse prevention. The emergence of new regimens presents a promising avenue for enhancing treatment options, emphasizing the ongoing evolution in the pursuit of more effective and accessible MDR-TB therapies.

Challenges and Limitations Associated With Long Regimens

Treatment success and relapse: Longer regimens have traditionally served as the cornerstone in the treatment of MDR-TB. However, a notable study revealed that rates of non-conversion or reversion of cultures, relapse, and death were somewhat elevated in the shorter regimen arm, although statistical significance was not reached [8]. Remarkably, the study underscored the protective role of treatment prolongation against relapse, emphasizing the critical importance of the total duration of treatment in ensuring sustained therapeutic success [6].

Standardization and resistance: The standardization of shorter regimens, as the World Health Organization (WHO) recommends, presents challenges when confronted with resistance to its constituent medications. The WHO explicitly advises against using the shorter regimen in the presence of resistance to any of its component drugs, shedding light on the potential limitations imposed by resistance profiles on treatment standardization [8].

Eligibility and uncertainties: The eligibility criteria for the shorter regimen demand a high likelihood of susceptibility to its component drugs and the absence of prior treatment with second-line drugs. Despite these criteria, uncertainties persist regarding the comparative effectiveness of the shorter regimen when weighed against individualized longer regimens, raising questions about its applicability and efficacy across diverse patient populations [8].

Monitoring and component selection: A fundamental shift in the monitoring approach is recommended, advocating for the use of cultures rather than relying solely on clinical parameters to evaluate the efficacy of MDR-TB regimens. The selection of medications for longer regimens adheres to a priority order based on revised guidelines, emphasizing incorporating at least four drugs likely to be effective in the initial six months and three drugs thereafter [5]. These guidelines address the multifaceted challenges associated with long regimens for MDR-TB treatment, including concerns about treatment success, relapse, standardization, resistance, eligibility, uncertainties, and the imperative need for meticulous monitoring and component selection. Together, these factors illuminate the intricate nature of MDR-TB management, emphasizing the ongoing considerations and complexities in navigating the optimal therapeutic approach.

Adverse Effects and Tolerability

The treatment of MDR-TB imposes a substantial burden of adverse drug reactions (ADRs) due to the prolonged duration, intricate nature, and inherent toxicity of the prescribed regimens. Numerous studies have documented diverse ADRs associated with MDR-TB therapy, encompassing symptoms such as nausea, vomiting, arthralgia, psychiatric disturbances, gastritis, hearing disturbance, skin rashes, headache, peripheral neuropathy, visual disturbance, gastrointestinal disturbances, psychiatric disorders, hepatitis, hypothyroidism, epileptic seizures, dermatological effects, ototoxicity, and nephrotoxicity [12-14]. The manifestation of ADRs can have significant implications and may influence therapy outcomes, underscoring the importance of proper management. An examination of 788 MDR-TB patients in India found no substantial correlation between ADRs and adverse treatment outcomes like death, loss to follow-up, transfer out, and switching to extensively drug-resistant TB (XDR-TB) [12]. However, ADRs can prompt therapy interruption, emphasizing the critical need for effective management strategies to safeguard patient well-being [12].

The frequency of ADRs during MDR-TB treatment can be considerable, with a notable proportion of patients encountering at least one ADR throughout therapy [13]. Managing ADRs stands as an indispensable facet of MDR-TB therapy, and the elevated incidence of these reactions underscores the imperative for robust strategies to mitigate their impact on patient well-being and treatment outcomes. The treatment of MDR-TB is intricately linked with a heightened occurrence of ADRs, encompassing a wide array of symptoms affecting diverse organ systems. The meticulous management of these ADRs is pivotal to ensuring patient well-being and optimizing treatment outcomes.

Patient Adherence and Compliance

The successful treatment of MDR-TB hinges on patient adherence and compliance, with various factors influencing these crucial components. Demographic factors, socioeconomic status, health insurance, and the potential impact of treatment side effects are key determinants [15-17]. A study conducted in Chongqing, China, highlighted that patients aged 55 or older, migrants, those lacking prior MDR-TB case management, unmarried individuals, and females were associated with poor adherence behavior (p<0.05) [17]. In Indonesia, a study identified a positive correlation between health insurance and medication adherence, noting that TB patients with high medication adherence exhibited a more favorable quality of life [16].

In Turkey, a study revealed that 55% of MDR-TB patients demonstrated compliance with treatment, with women exhibiting higher adherence rates (85%) compared to men (64%) [15]. Notably, 45% of patients experienced medication side effects or drug toxicity, a factor strongly associated with lower adherence [15]. A non-inferiority randomized controlled trial compared adherence to MDR-TB treatment between patients on self-administered therapy (SAT) and those under directly observed therapy (DOT) [18]. Surprisingly, the study found similar adherence rates between the two groups, suggesting that SAT could serve as a viable alternative approach to MDR-TB treatment [18]. The collective evidence underscores the pivotal role of patient adherence and compliance in the success of MDR-TB treatment. Tailoring strategies to enhance adherence should consider the unique needs and circumstances of MDR-TB patients, taking into account demographic variables, socioeconomic factors, health insurance coverage, and the mitigation of treatment-related side effects. Such patient-centered approaches are essential for optimizing treatment outcomes in the challenging landscape of MDR-TB.

Short regimens in MDR-TB treatment

Definition and Components of Short Regimens

Recent research and WHO guidelines have brought shorter regimens to the forefront of attention in the treatment of MDR-TB. Typically designed with minimal second-line TB medicines deemed effective based on patient history or drug-resistance testing, these regimens aim to streamline treatment approaches [4]. The World Health Organization (WHO) advocates for a standardized shorter regimen (STR) for MDR-TB, featuring a treatment duration of nine to 12 months, with the overarching goals of cost reduction, improved compliance, and heightened cure rates [4,19]. The STR conventionally encompasses an intensive phase lasting four to six months and a continuation phase spanning five to six months. While associated with a lower risk of loss to follow-up, it carries a heightened risk of failure or relapse in cases of resistance to component drugs [20]. Comprising second-line drugs such as kanamycin, moxifloxacin, prothionamide, and others, the STR is subject to eligibility requirements demanding a high likelihood of susceptibility and no prior treatment with second-line drugs [21]. Challenges in implementing shorter regimens for MDR-TB treatment include issues related to the availability and procurement of new medications and the monitoring of clinical outcomes [22]. Despite the promise exhibited by shorter regimens, it is imperative to consider potential challenges and the risk of resistance to component drugs when translating these approaches into clinical practice [4,20,22].

Development and Rationale for Short Regimens

The impetus behind the development of short regimens for the treatment of MDR-TB stems from the imperative to devise more effective, safe, and concise treatment options. Research has prominently centered around the empirical assessment of short-course regimens, particularly those featuring bedaquiline (BDQ), with the primary goal of mitigating drug resistance development and enhancing treatment outcomes [22]. The prolonged duration and associated challenges inherent in traditional MDR-TB treatment, coupled with suboptimal success rates, have underscored the pressing need for briefer yet more potent regimens [23].

Acknowledging these imperatives, the World Health Organization (WHO) has endorsed the conditional use of six-month all-oral regimens for MDR-TB. This endorsement is geared toward minimizing treatment toxicity and maximizing efficacy, with a strategic aim to address challenges related to patient adherence, diminish the risk of nonadherence and loss of follow-up, and elevate overall treatment success rates for MDR-TB [24]. Ongoing research endeavors are actively seeking to identify the optimal combinations and doses of drugs for inclusion in these treatment-shortening regimens [20]. The advent of shortened regimens represents a significant leap forward, poised to revolutionize the care provided to individuals grappling with MDR-TB. However, persistent challenges tied to drug availability, potential toxicity concerns, and the practical implementation of these regimens remain integral aspects that necessitate ongoing attention and investigation [24]. Despite these challenges, the development and endorsement of shortened regimens mark a crucial step forward in the pursuit of more efficient and patient-friendly solutions for MDR-TB treatment.

Comparative Efficacy of Short Regimens vs. Long Regimens

The comparative effectiveness of short regimens versus long regimens in the treatment of MDR-TB has been a focal point of research. A systematic review and meta-analysis revealed that the shorter regimen exhibited higher treatment success rates compared to longer regimens (pooled proportions 80.0% versus 75.3%), primarily attributed to reduced instances of loss to follow-up [8]. However, it is crucial to highlight that the shorter regimen was associated with a heightened risk of failure or relapse when resistance to component drugs was present [8]. Employing multivariable analyses to adjust for potential confounding variables is imperative when comparing the efficacy of standardized shorter regimens to individualized regimens of a longer composition, aligning with WHO guidelines for MDR-TB treatment [8]. Ongoing research endeavors aim to discern the optimal drug combinations and doses for inclusion in treatment-shortening regimens. Simultaneously, efforts are directed toward addressing challenges related to patient adherence and the development of drug resistance [20,23]. These initiatives underscore the dynamic nature of MDR-TB treatment strategies, emphasizing the continuous pursuit of refined approaches that balance efficacy, safety, and patient adherence in the quest for optimal outcomes.

Safety Profile and Tolerability

The safety profile and tolerability of shorter regimens for the treatment of MDR-TB have been scrutinized in various studies. A study conducted in Uzbekistan revealed skepticism among healthcare providers regarding the safety of the shorter regimen [25]. Concerns about the regimen's potential toxicity have also been expressed [25]. However, a systematic review and meta-analysis counterbalanced these concerns, indicating that the shorter regimen was associated with a lower risk of loss to follow-up compared to longer regimens [26]. Evaluating the effectiveness and safety of novel shorter MDR-TB regimens through adverse event rates remains a crucial secondary objective in ongoing research [27].

While concerns have been raised about the safety and tolerability of shorter regimens, the available evidence generally suggests that they are safe and well-tolerated. It is important to note that further research is needed to comprehensively evaluate these regimens' safety profile, including determining optimal doses and identifying drug combinations suitable for inclusion in treatment-shortening regimens [20,25,26]. The ongoing exploration of these aspects will contribute to refining our understanding of the safety considerations associated with shorter regimens for MDR-TB treatment.

Implementation Challenges and Considerations

Resistance to component drugs: Shorter regimens present a potential challenge in terms of a higher risk of treatment failure or relapse when there is resistance to the component drugs [28]. This underscores the importance of carefully assessing drug resistance profiles before implementing these regimens to optimize treatment outcomes.

Availability and procurement of new medications: The successful implementation of shorter regimens may hinge on the availability and procurement of new medications, particularly in resource-limited settings where challenges in obtaining these drugs may arise [29]. Overcoming these logistical hurdles is crucial for the widespread adoption of shorter regimens.

Monitoring clinical outcomes: The effectiveness of shorter regimens necessitates vigilant monitoring of clinical outcomes, which may require additional resources and infrastructure. This monitoring is essential for assessing the impact of these regimens and ensuring their efficacy in diverse patient populations.

Patient adherence: Shorter regimens may carry a higher risk of nonadherence and loss of follow-up, potentially impacting treatment success rates [28]. Implementing strategies to enhance patient adherence, such as robust education and support programs, is imperative for optimizing the effectiveness of shorter regimens.

Healthcare providers' doubts: The doubts among healthcare providers regarding the safety of shorter regimens can pose a challenge, influencing their willingness to prescribe and implement these regimens in clinical practice [28]. Addressing these concerns through comprehensive training and evidence-based communication is essential for fostering confidence in the medical community.

Cost-effectiveness: Shorter regimens may face challenges related to cost-effectiveness, especially when compared to longer regimens. This cost barrier can be particularly pronounced in resource-limited settings, necessitating careful consideration of the economic feasibility of implementing shorter regimens [22].

Patient Adherence and Compliance

Patient education and counseling: Providing comprehensive patient education and counseling is essential for ensuring that individuals thoroughly understand the disease process and are well-informed about the associated risks and benefits of treatment adherence [30]. This involves empowering patients with the knowledge necessary to participate actively in their treatment plan.

Incentives and enablers: Interventions incorporating incentives and enablers are crucial in promoting treatment adherence. These may include financial or material rewards that motivate patients and interventions addressing barriers like cost, distance, and medication availability [30]. By addressing these practical concerns, patients are better positioned to adhere to their treatment regimens.

Reminders and tracers: Implementing reminder systems and tracers is instrumental in enhancing treatment adherence. This involves deploying interventions that remind patients to take medications or attend appointments and follow-up contacts after instances of non-adherence to improve subsequent adherence to treatment [30]. These strategies help reinforce the importance of adherence throughout treatment.

Digital technologies: Leveraging digital technologies, such as short message services (SMS) via mobile phones and video-observed therapy (VOT), represents a contemporary approach to supporting treatment adherence [30]. These technologies offer innovative ways to connect with patients, providing timely reminders and facilitating remote observation of medication intake. In a study assessing outcomes and adherence to the shorter MDR-TB regimen, the rate of treatment non-compliance among study subjects was reported at 25.7% [2]. Notably, incorporating adherence interventions into DOT was associated with favorable outcomes, including reduced mortality and loss to follow-up, along with higher rates of treatment success and cure [30]. This highlights the effectiveness of multifaceted interventions in improving adherence and overall treatment outcomes in MDR-TB cases.

Programmatic management of drug-resistant tuberculosis (PMDT)

Overview of PMDT

The PMDT is pivotal in providing appropriate treatment for MDR-TB and constitutes a crucial component of global TB control. According to the World Health Organization (WHO), an estimated 480,000 new cases of MDR-TB emerged in 2014, with 8.7% of these cases classified as XDR-TB [31]. PMDT encompasses diverse elements, including laboratory support, treatment strategies, program-relevant research, epidemiology, and the management of contacts [31].

In the Indian context, PMDT services were introduced in 2007, achieving complete geographic coverage by 2013. The PMDT guidelines in India undergo regular updates, focusing on emerging diagnostic trends, new drugs, and therapeutic approaches. This includes the incorporation of oral shorter regimens featuring bedaquiline (Bdq) and refined definitions for pre-XDR and XDR [32].

PMDT involves the execution of treatment regimens, and recent studies have compared the effectiveness of individualized LTR and standardized STR. These investigations reveal that STR demonstrates superior antimicrobial activity against MDR-TB, leading to significantly earlier treatment completion when contrasted with LTR [33]. The comprehensive nature of PMDT addresses the challenges posed by MDR-TB and XDR-TB, with continuous updates to its guidelines and regimens reflecting the latest evidence and best practices. In essence, PMDT stands as a vital approach to managing drug-resistant TB, tackling various facets of treatment, research, and epidemiology in response to the evolving landscape of MDR-TB and XDR-TB.

Key Components and Strategies

Situational analysis: A comprehensive situational analysis is crucial for understanding the landscape of MDR-TB. This involves assessing the burden of TB, available resources, and the existing model of care and identifying gaps that need to be addressed [34]. This analysis serves as the foundational step in tailoring strategies to the specific needs and challenges of the MDR-TB context.

Planning matrix: Developing a planning matrix is essential for outlining strategic objectives and activities related to the management of MDR-TB. This matrix is a structured framework to guide decision-making and resource allocation, ensuring a systematic and goal-oriented approach [34].

Budget estimation: Estimating the budget required for PMDT is a critical aspect of program planning. This includes a detailed breakdown of costs, encompassing second-line drugs, ancillary drugs, patient/DOTS provider support, and patient follow-up. Accurate budget estimation is fundamental for securing financial resources [34].

Operational plan: Developing an operational plan is essential for translating strategic objectives into actionable steps. This plan includes key elements such as background information, strategic objectives, roles and responsibilities, detailed activities, and allocating necessary resources. An effective operational plan provides a roadmap for the successful implementation of PMDT [34].

Monitoring and evaluation plan: Creating a robust monitoring and evaluation plan is vital for assessing the ongoing effectiveness of PMDT programs. This plan includes the development of a Monitoring and Evaluation (M&E) logical framework outlining key indicators, data collection methods, and evaluation timelines. Regular monitoring and evaluation are critical for identifying successes, challenges, and areas for improvement [34].

Technical assistance plan: Developing a technical assistance plan is geared towards supporting implementing PMDT. This involves outlining specific areas where technical assistance is needed, identifying potential sources of support, and establishing mechanisms for ongoing assistance. A well-crafted technical assistance plan enhances the program's capacity and effectiveness [34].

Role of PMDT in Long Regimens

The PMDT is pivotal in managing extended drug-resistant TB regimens. PMDT encompasses the implementation of treatment protocols, and recent research has delved into the comparative effectiveness of individualized LTR against standardized STR. These studies indicate that STR exhibits heightened antimicrobial activity against MDR-TB, leading to significantly earlier completion of treatment compared to LTR [3].

In the Indian context, PMDT services were introduced in 2007, achieving comprehensive geographic coverage by 2013. The guidelines for PMDT in India undergo regular updates, concentrating on emerging diagnostic trends, novel drugs, and therapeutic approaches. This includes the incorporation of oral shorter regimens featuring bedaquiline (Bdq) and refined definitions for pre-XDR and XDR [35]. The 2019 iteration of the PMDT guidelines in India integrates the use of the shorter MDR TB regimen and an all-oral longer MDR TB regimen with new drugs under RNTCP [36]. PMDT plays a crucial role in overseeing extended regimens for drug-resistant tuberculosis (TB), emphasizing the implementation of effective treatment protocols and the continual updating of guidelines to align with emerging trends and best practices.

Role of PMDT in Short Regimens

PMDT assumes a crucial role in facilitating the implementation of shorter regimens for the treatment of MDR-TB. Within the Indian context, the PMDT guidelines have seamlessly incorporated the utilization of the shorter MDR-TB regimen and an all-oral longer MDR-TB regimen featuring new drugs under the Revised National Tuberculosis Control Program (RNTCP) [36]. The 2019 guidelines specifically recommend an all-oral longer regimen with new drugs for the treatment of MDR/RR-TB [36].

Since its initiation in 2008, PMDT services have been consistently rolled out, achieving comprehensive geographic coverage by 2013 [37]. Beyond its role in MDR-TB treatment, PMDT extends its influence to the evaluation of strategies and progress concerning the management of latent TB [37]. This underscores the instrumental role PMDT plays in not only integrating and implementing shorter regimens for MDR-TB treatment but also in continually assessing strategies and advancements in the realm of drug-resistant TB management.

Challenges and Opportunities in PMDT Implementation

The implementation of the PMDT faces several challenges and opportunities as described in Table 1.

**Table 1:** Challenges and opportunities in PMDT implementation

Challenges	Opportunities
Resistance to component drugs in the shorter regimens can result in treatment failure or relapse [34].	Shorter regimens for MDR-TB treatment have shown better treatment success and reduced loss to follow-up compared to longer regimens [38].
The availability and procurement of new medications, as well as monitoring clinical outcomes, may pose challenges in implementing shorter regimens [39].	Shorter regimens for MDR-TB treatment could potentially reduce treatment costs, making it more affordable for resource-limited settings [38].
Adverse events and their management will also be important considerations in implementing shorter regimens for MDR-TB treatment [39].	Shorter regimens for MDR-TB treatment result in significantly earlier treatment completion, which could improve patient outcomes and reduce the burden on healthcare systems [38].
Human resources and their development remain a major cross-cutting issue for both the public and private sectors, relating not just to adequate numbers of staff but also their job responsibilities and capacity [40].	Decentralized PMDT implementation can help address issues such as treatment default and improve access to care for MDR-TB patients [38].

Comparative analysis and synthesis

Effectiveness and Efficiency of Long vs. Short Regimens

The comparative effectiveness and efficiency of long versus short regimens for MDR-TB have been extensively investigated. Since 2016, the World Health Organization (WHO) has endorsed standardized shorter regimens (STR) for MDR-TB treatment, aiming to achieve cost reduction, enhance compliance, and improve cure rates [41]. These STRs, designed to last nine to 12 months, have demonstrated superior treatment success and reduced rates of loss to follow-up when compared to individualized LTR [8]. In a study comparing STR with LTR, the former exhibited heightened antimicrobial activity against MDR-TB, leading to significantly earlier treatment completion [33]. However, it is essential to acknowledge that STRs carry a higher risk of failure or relapse in the presence of resistance to component drugs [8].

The implementation of shorter regimens for MDR-TB treatment necessitates careful consideration of factors such as the availability and procurement of new medications, the monitoring of clinical outcomes, and the management of adverse events [24]. Despite the promise shown by shorter regimens in terms of effectiveness and efficiency, it is imperative to acknowledge the challenges and potential resistance to component drugs. These factors should be thoroughly assessed when determining the most suitable regimen for MDR-TB treatment.

Impact on Treatment Outcomes

Various studies have delved into the treatment outcomes of patients facing MDR-TB. In one study, it was reported that after 24 months, 69% of patients successfully completed their treatment, while 27% unfortunately succumbed to the disease [42]. Another study highlighted a higher treatment success rate with the shorter regimen (80.0%) compared to longer regimens (75.3%), primarily attributed to a lower incidence of loss to follow-up [8]. In Ghana, a study demonstrated that over two-thirds of patients (71.7%) achieved a successful outcome in MDR-TB treatment, but with a mortality rate of 17.0% [43]. Additionally, a retrospective cohort study in China disclosed that 24.9% of patients necessitated a change in MDR-TB treatment due to adverse events, although the treatment success rate was unspecified [44]. Overall, the evidence indicates that the effectiveness of the shorter regimen is comparable to or even superior to longer regimens, marked by lower loss to follow-up. However, potential challenges such as adverse events should be vigilantly managed [8,43,44].

Cost-Effectiveness and Resource Utilization

Several studies have undertaken an examination of the cost-effectiveness and resource utilization in the context of long versus short regimens for the treatment of MDR-TB. A systematic review identified a bedaquiline-based regimen as cost-effective for MDR-TB [45]. In Ethiopia, another study determined that the cost per successfully treated HIV-negative patient for MDR-TB was $8,416 at a treatment initiative center and $6,657 at a treatment follow-up center [46]. In India, decentralized MDR-TB care was estimated to save patients up to $1,666.50 per case, with an incremental cost-effectiveness ratio of $2,382.68 per quality-adjusted life year gained [47]. However, it is important to note that MDR-TB treatment entails greater resource utilization and higher costs compared to non-MDR-TB treatment [48].

Despite the documented success and reduced loss to follow-up associated with shorter regimens in MDR-TB treatment, careful consideration of the cost-effectiveness and resource utilization of these regimens is crucial, particularly in low- and middle-income countries where resources may be constrained [45-47].

Considerations for Specific Patient Populations

The treatment of MDR-TB demands special considerations for specific patient populations, including children and those with comorbidities. Recent advancements in regimens, particularly the introduction of newer, shorter protocols, have substantially improved the manageability of treatment for these patients, offering a stark contrast to earlier regimens characterized by a high pill burden and severe side effects [49]. The distinctive impact of MDR-TB on various patient groups is evident in treatment outcomes. A study reported a 69% composite treatment success rate for MDR-TB patients, accompanied by a 27% death rate at the 24-month mark [42]. This underscores the challenges posed by MDR-TB, showcasing lower cure rates and heightened resource utilization, resulting in significantly elevated healthcare costs [48].

In the specific context of patient populations, a retrospective cohort study in China highlighted that 24.9% of MDR-TB patients required a change in treatment due to adverse events. This emphasizes the critical importance of closely monitoring and managing treatment for individuals within these groups [44]. While newer, shorter regimens hold promise in enhancing treatment outcomes and alleviating the burden on patients, it remains imperative to evaluate their impact on distinct patient populations, including children and those with comorbidities. Moreover, the associated resource utilization and potential adverse events should be carefully considered.

Future directions and research gaps

Emerging Therapies and Treatment Approaches

Treatment success discrepancy: A comprehensive review of global treatment outcomes for drug-resistant TB has illuminated a noteworthy trend - although there has been an increase in treatment success rates for MDR/RR-TB, these rates still fall below the World Health Organization's (WHO) targeted benchmark of 90% [50]. This disparity underscores a substantial gap in achieving the desired treatment success rates, signaling an urgent need for strategic interventions and improvements.

Challenges of prolonged treatment: The challenges inherent in the treatment of MDR-TB are multifaceted. Prolonged treatment courses, the intricacy of daily regimens, reliance on injectable drugs, and the occurrence of adverse reactions collectively pose significant hurdles [50]. These challenges not only complicate the treatment process but also elevate the risk of non-adherence, ultimately influencing treatment outcomes. Addressing these complexities is crucial for enhancing the overall effectiveness of MDR-TB treatment.

Cost and adverse events: The financial implications of MDR/RR-TB treatment are profound, exerting potentially catastrophic effects on both affected individuals and healthcare systems [50]. The associated costs serve as a formidable barrier, hindering access to essential resources needed for effective treatment. Additionally, the incidence of adverse events during the course of treatment is a critical consideration that can significantly impact patient outcomes [33]. Strategizing effective management strategies for both cost and adverse events is essential for optimizing the overall treatment experience.

Research focus: Recognizing the challenges posed by prolonged regimens and the escalating threat of resistance, there is a discernible shift in research focus towards developing shorter, all-oral regimens for drug-resistant TB [50]. This shift aims to improve patient care, reduce the duration of treatment, and curb the spread of resistance. Ongoing clinical trials are actively evaluating the effectiveness and safety of these shorter regimens, representing a pivotal step in reshaping the landscape of MDR-TB treatment and advancing patient-centric approaches [50].

Advancements in Diagnostics and Monitoring

Recent strides in diagnosing and treating MDR-TB have yielded various diagnostic techniques, encompassing both phenotypic and molecular approaches, aimed at swiftly identifying MDR-TB strains in suspected patients [51]. Over the past decade, molecular diagnostics for detecting Mycobacterium tuberculosis complex and predicting drug resistance have been integrated, expediting TB diagnosis and enhancing case detection [52]. Recognizing these advancements, the World Health Organization (WHO) has endorsed the use of rapid molecular tests for diagnosing MDR-TB since 2015 [52].

Despite these breakthroughs, challenges persist in ensuring timely, precise, and cost-effective diagnostics, particularly in resource-limited settings where access to power, equipment, and technical expertise is constrained [53]. Ongoing extensive research is dedicated to devising accurate and prompt methods for detecting drug resistance, even in resource-poor settings [53]. While considerable headway has been achieved in crafting diagnostic tools for MDR-TB, an ongoing imperative exists for accessible and efficacious diagnostic approaches, particularly in resource-limited settings, to augment the detection of drug-resistant TB and elevate the standard of patient care.

Strategies for Improving Patient Engagement and Adherence

Patient-centered care: A pivotal aspect involves adopting a patient-centered approach that recognizes the intricate interplay of clinical, behavioral, social, and economic factors influencing adherence [54]. This approach necessitates a team-based, decentralized care model, requiring substantial investment in human resources. The goal is to establish high-quality, interconnected networks of social support to better cater to the diverse needs of patients [54].

Adherence interventions: Various proven adherence interventions contribute significantly to improved TB treatment outcomes [30]. These interventions encompass a range of strategies, including patient education and counseling, incentives and enablers, psychological support, and the integration of digital technologies. Importantly, these interventions should be tailored to meet the specific needs of each patient. Delivery channels may include mobile phones, VOT, and home visits by community health workers [55].

Risk factor identification: A critical step in developing effective interventions involves the identification of risk factors associated with poor engagement in MDR-TB care [56]. Factors such as missed appointments, treatment interruption, sub-optimal medication adherence, and loss of follow-up must be recognized and addressed systematically. Consistent risk factors, such as male sex and younger age, have been identified as predictors of poor engagement [56].

Self-administered therapy: A non-inferiority randomized controlled trial has demonstrated that SAT is as effective as DOT in terms of adherence rates among MDR-TB patients [18]. SAT presents a more feasible and acceptable option for patients facing challenges associated with DOT, such as transportation constraints and time limitations [18].

Areas Requiring Further Investigation and Research

Shorter treatment regimens: Despite the promising outlook of shorter regimens in treating MDR-TB, extensive research is imperative to assess their effectiveness, safety, and tolerability across diverse patient populations [57]. Moreover, there is a need to explore the optimal treatment duration and investigate the potential role of adjunctive therapies, such as immunomodulators, in enhancing treatment outcomes [57].

Vaccine development: The development of a robust TB vaccine remains a pivotal focus in research endeavors [57]. Ongoing clinical trials are assessing various vaccine candidates, necessitating further research to comprehensively evaluate their safety, efficacy, and potential impact on TB control efforts [57].

Contact tracing: Integral to TB control, contact tracing requires further research to optimize its efficacy in identifying and treating cases of MDR-TB [58]. This involves evaluating the yield of contact tracing, assessing the effectiveness of preventive therapy, and scrutinizing the influence of social and behavioral factors on contact tracing outcomes [58].

Drug discovery: The ongoing battle against drug resistance mandates continuous research efforts for the discovery of novel drugs and drug combinations for MDR-TB treatment [57]. There is a need to identify new drug targets, refine drug dosing and delivery methods, and rigorously evaluate the safety and efficacy of emerging drug candidates [57].

## Conclusions

In conclusion, this review provides a comprehensive examination of the treatment strategies for MDR-TB within the framework of PMDT. The analysis of long and short regimens reveals nuanced findings, highlighting the need for a tailored and patient-centric approach in clinical practice. The implications for healthcare practitioners underscore the importance of considering factors such as drug-resistance patterns, comorbidities, and treatment adherence when choosing between long and short regimens. Recommendations for PMDT programs emphasize the integration of advanced diagnostics, continuous surveillance, and ongoing training for healthcare professionals to optimize patient outcomes. The overall outlook on long versus short regimens reflects the complexities of balancing treatment efficacy with patient-friendly approaches. The review encourages a balanced perspective, recognizing the strengths and weaknesses of both strategies and calling for continued advancements in anti-TB medications and diagnostics. As the field evolves, the collaborative efforts of national health agencies, international organizations, and local communities remain pivotal in refining PMDT programs and improving the outlook for individuals facing the challenges of MDR-TB.

## References

[1] Seung KJ, Keshavjee S, Rich ML (2015). Multidrug-resistant tuberculosis and extensively drug-resistant tuberculosis. Cold Spring Harb Perspect Med.

[2] Kumari SL, Kongara S, Bhaskar K, Srikanti R, Bhushana Rao CR, Sanjana PH (2023). Outcomes and adherence of shorter MDR TB regimen in patients with multidrug resistant tuberculosis. Indian J Tuberc.

[3] World Health Organization (2014). Companion Handbook to the WHO Guidelines for the Programmatic Management of Drug-Resistant Tuberculosis. World Health Organization.

[4] World Health Organization (2019). Key definitions. In:. WHO Consolidated Guidelines on Drug-Resistant Tuberculosis Treatment.

[5] (2024). Choice of components for the longer MDR-TB regimens. https://tbksp.org/en/node/2520.

[6] Mase SR, Chorba T (2019). Treatment of drug-resistant tuberculosis. Clin Chest Med.

[7] (2024). Tuberculosis: key changes to the treatment of drug-resistant TB. https://www.who.int/news-room/questions-and-answers/item/tuberculosis-key-changes-to-the-treatment-of-drug-resistant-tb.

[8] Abidi S, Achar J, Assao Neino MM (2020). Standardised shorter regimens versus individualised longer regimens for rifampin- or multidrug-resistant tuberculosis. Eur Respir J.

[9] Cox HS, Morrow M, Deutschmann PW (2008). Long term efficacy of DOTS regimens for tuberculosis: systematic review. BMJ.

[10] Phillips PP, Van Deun A, Ahmed S (2020). Investigation of the efficacy of the short regimen for rifampicin-resistant TB from the STREAM trial. BMC Med.

[11] (2024). Landmark clinical trial redefines multidrug-resistant tuberculosis treatment options. https://www.pih.org/article/landmark-clinical-trial-redefines-multidrug-resistant-tuberculosis-treatment-options.

[12] Ausi Y, Santoso P, Sunjaya DK, Barliana MI (2021). Between curing and torturing: burden of adverse reaction in drug-resistant tuberculosis therapy. Patient Prefer Adherence.

[13] Khan FU, Khan A, Khan FU (2022). Assessment of adverse drug events, their risk factors, and management among patients treated for multidrug-resistant TB: a prospective cohort study from Pakistan. Front Pharmacol.

[14] Yang TW, Park HO, Jang HN (2017). Side effects associated with the treatment of multidrug-resistant tuberculosis at a tuberculosis referral hospital in South Korea: a retrospective study. Medicine (Baltimore).

[15] Khomova N, Tashpulatova F, Sultanov S (2017). Compliance - is patient adherence to treatment, as well as partnerships between doctor and patient. Eur Respir J.

[16] Yani DI, Juniarti N, Lukman M (2022). Factors related to complying with anti-tb medications among drug-resistant tuberculosis patients in indonesia. Patient Prefer Adherence.

[17] Xing W, Zhang R, Jiang W (2021). Adherence to multidrug resistant tuberculosis treatment and case management in Chongqing, China - a mixed method research study. Infect Drug Resist.

[18] Wekesa C, Sekaggya-Wiltshire C, Muyanja SZ, Lume I, Nabaggala MS, Parkes-Ratanshi R, Akello SA (2023). Comparing adherence to MDR-TB treatment among patients on self-administered therapy and those on directly observed therapy: non-inferiority randomized controlled trial. Trials.

[19] Falzon D, Schünemann HJ, Harausz E, González-Angulo L, Lienhardt C, Jaramillo E, Weyer K (2017). World Health Organization treatment guidelines for drug-resistant tuberculosis, 2016 update. Eur Respir J.

[20] Grace AG, Mittal A, Jain S, Tripathy JP, Satyanarayana S, Tharyan P, Kirubakaran R (2019). Shortened treatment regimens versus the standard regimen for drug-sensitive pulmonary tuberculosis. Cochrane Database Syst Rev.

[21] Tsang CA, Shah N, Armstrong LR, Marks SM (2020). Eligibility for a shorter treatment regimen for multidrug-resistant tuberculosis in the united states, 2011-2016. Clin Infect Dis.

[22] Doan TN, Cao P, Emeto TI, McCaw JM, McBryde ES (2018). Predicting the outcomes of new short-course regimens for multidrug-resistant tuberculosis using intrahost and pharmacokinetic-pharmacodynamic modeling. Antimicrob Agents Chemother.

[23] Silva DR, Mello FC, Migliori GB (2020). Shortened tuberculosis treatment regimens: what is new?. J Bras Pneumol.

[24] Sinha P, Jacobson KR, Horsburgh CR Jr, Acuña-Villaorduña C (2023). At long last: short, all-oral regimens for multidrug-resistant tuberculosis in the United States. Open Forum Infect Dis.

[25] Trubnikov A, Hovhannesyan A, Akopyan K (2021). Effectiveness and safety of a shorter treatment regimen in a setting with a high burden of multidrug-resistant tuberculosis. Int J Environ Res Public Health.

[26] Nyang’wa B-T, Berry C, Kazounis E (2023). Short oral regimens for pulmonary rifampicin-resistant tuberculosis (TB-PRACTECAL): an open-label, randomised, controlled, phase 2B-3, multi-arm, multicentre, non-inferiority trial. Lancet Respir Med.

[27] Ahmad Khan F, Salim MA, du Cros P (2017). Effectiveness and safety of standardised shorter regimens for multidrug-resistant tuberculosis: individual patient data and aggregate data meta-analyses. Eur Respir J.

[28] Liu Q, Sun F, Li Y, Bao J, Zhang Y, Zhang W (2018). Practical considerations to implement the shorter regimen to MDR-TB patients in China. Clin Microbiol Infect.

[29] Institute of Medicine (US) (2012). Facing the Reality of Drug-Resistant Tuberculosis in India: Challenges and Potential Solutions. Medicine, the Indian National Science Academy, and the Indian Council of Medical Research. National Academies Press.

[30] Alipanah N, Jarlsberg L, Miller C, Linh NN, Falzon D, Jaramillo E, Nahid P (2018). Adherence interventions and outcomes of tuberculosis treatment: a systematic review and meta-analysis of trials and observational studies. PLoS Med.

[31] Mitnick CD, Rodriguez CA, Hatton ML (2016). Programmatic management of drug-resistant tuberculosis: an updated research agenda. PLoS One.

[32] Gupta M, Ish P, Malhotra N (2022). Recent updates in diagnosis and management of drug-resistant tuberculosis in India: a paradigm shift and the way ahead during the COVID-19 crisis. Indian J Tuberc.

[33] Wahid A, Ghafoor A, Khan AW (2022). Comparative effectiveness of individualized longer and standardized shorter regimens in the treatment of multidrug resistant tuberculosis in a high burden country. Front Pharmacol.

[34] Jang JG, Chung JH (2020). Diagnosis and treatment of multidrug-resistant tuberculosis. Yeungnam Univ J Med.

[35] (2024). PMDT: central TB division. https://tbcindia.gov.in/index1.php.

[36] Chaudhuri AD (2020). Recent changes in guidelines on programmatic management of drug resistant tuberculosis in India 2019: a paradigm shift in tuberculosis control. J Assoc Chest Physicians.

[37] (2024). Programmatic management of drug-resistant tuberculosis. https://iris.who.int/bitstream/handle/10665/205664/B4953.pdf?sequence=1.

[38] Hoang TT, Nguyen NV, Dinh SN (2015). Challenges in detection and treatment of multidrug resistant tuberculosis patients in Vietnam. BMC Public Health.

[39] Dholakia Y, Mistry N (2018). Challenges and opportunities for programmatic management of drug resistant tb in India. SM Trop Med J.

[40] Daley CL (2019). Global scale-up of the programmatic management of multidrug-resistant tuberculosis. Indian J Tuberc.

[41] Prasad R, Gupta N, Banka A (2017). Shorter &amp; cheaper regimen to treat multidrug-resistant tuberculosis: a new hope. Indian J Med Res.

[42] Wakjira MK, Sandy PT, Mavhandu-Mudzusi AH (2022). Treatment outcomes of patients with MDR-TB and its determinants at referral hospitals in Ethiopia. PLoS One.

[43] Panford V, Kumah E, Kokuro C (2022). Treatment outcomes and associated factors among patients with multidrug-resistant tuberculosis in Ashanti Region, Ghana: a retrospective, cross-sectional study. BMJ Open.

[44] Lecai J, Mijiti P, Chuangyue H, Qian G, Weiguo T, Jihong C (2023). Treatment outcomes of multidrug-resistant tuberculosis patients receiving ambulatory treatment in Shenzhen, China: a retrospective cohort study. Front Public Health.

[45] Byun JY, Kim HL, Lee EK, Kwon SH (2021). A systematic review of economic evaluations of active tuberculosis treatments. Front Pharmacol.

[46] Alemayehu S, Yigezu A, Hailemariam D, Hailu A (2020). Cost-effectiveness of treating multidrug-resistant tuberculosis in treatment initiative centers and treatment follow-up centers in Ethiopia. PLoS One.

[47] John D, Chatterjee P, Murthy S, Bhat R, Musa BM (2018). Cost effectiveness of decentralised care model for managing MDR-TB in India. Indian J Tuberc.

[48] Miller TL, Cirule A, Wilson FA (2013). The value of effective public tuberculosis treatment: an analysis of opportunity costs associated with multidrug resistant tuberculosis in Latvia. Cost Eff Resour Alloc.

[49] (2024). New data provide a boost for shorter drug-resistant TB regimens. https://www.cidrap.umn.edu/antimicrobial-stewardship/new-data-provide-boost-shorter-drug-resistant-tb-regimens.

[50] Saluzzo F, Adepoju VA, Duarte R, Lange C, Phillips PP (2023). Treatment-shortening regimens for tuberculosis: updates and future priorities. Breathe (Sheff).

[51] Ahmad S, Mokaddas E (2009). Recent advances in the diagnosis and treatment of multidrug-resistant tuberculosis. Respir Med.

[52] Günther G, Ruswa N, Keller PM (2022). Drug-resistant tuberculosis: advances in diagnosis and management. Curr Opin Pulm Med.

[53] Gill CM, Dolan L, Piggott LM, McLaughlin AM (2022). New developments in tuberculosis diagnosis and treatment. Breathe (Sheff).

[54] O'Donnell MR, Daftary A, Frick M (2016). Re-inventing adherence: toward a patient-centered model of care for drug-resistant tuberculosis and HIV. Int J Tuberc Lung Dis.

[55] Law S, Daftary A, O'Donnell M, Padayatchi N, Calzavara L, Menzies D (2019). Interventions to improve retention-in-care and treatment adherence among patients with drug-resistant tuberculosis: a systematic review. Eur Respir J.

[56] McNabb KC, Bergman A, Farley JE (2021). Risk factors for poor engagement in drug-resistant TB care in South Africa: a systematic review. Public Health Action.

[57] Bendre AD, Peters PJ, Kumar J (2021). Tuberculosis: past, present and future of the treatment and drug discovery research. Curr Res Pharmacol Drug Discov.

[58] Phyo AM, Kumar AM, Soe KT (2019). Contact investigation of multidrug-resistant tuberculosis patients: a mixed-methods study from Myanmar. Trop Med Infect Dis.

